# Effects of vaginal estrogen on serum estradiol during aromatase inhibitor therapy in breast cancer patients with vulvovaginal atrophy: a prospective trial

**DOI:** 10.1007/s10549-024-07564-8

**Published:** 2024-12-27

**Authors:** Mária Faltinová, Leena Vehmanen, Heli Lyytinen, Hanna Savolainen-Peltonen, Anni Virtanen, Mikko Haanpää, Esa Hämäläinen, Aila Tiitinen, Johanna Mattson

**Affiliations:** 1 Comprehensive Cancer Center, Helsinki University Hospital, University of Helsinki, PO Box 180, 00290 Helsinki, Finland; 2 Department of Obstetrics and Gynecology, Helsinki University Hospital and University of Helsinki, Helsinki, Finland; 3 HUSLAB, Helsinki University Hospital, Helsinki, Finland; 4 Department of Clinical Chemistry, University of Eastern Finland, Kuopio, Finland

**Keywords:** Aromatase inhibitor, Breast cancer, Estradiol, Intravaginal estrogen therapy, Liquid chromatography tandem mass spectrometry, Vulvovaginal atrophy

## Abstract

**Purpose:**

This study aimed to analyze changes in serum estradiol (E2) levels during concurrent vaginal estradiol therapy and adjuvant letrozole in postmenopausal breast cancer (BC) patients with vulvovaginal atrophy (VVA). Secondary objectives included assessing the effects of therapy on vaginal atrophy, quality of life (QoL) and menopause-related symptoms.

**Methods:**

20 postmenopausal patients undergoing adjuvant letrozole therapy and experiencing VVA symptoms were treated with vaginal estradiol for 12 weeks. Gynecologic examination and symptom screening were conducted at baseline and after 12 weeks. Serum E2 levels were analyzed at baseline, and at two, four, eight, and 12 weeks. E2 levels were measured using both a routine liquid chromatography-tandem mass spectrometry (LC–MS/MS) method and a highly sensitive (hsE2-MS) LC–MS/MS method.

**Results:**

At baseline, serum E2 levels, measured with hsE2-MS, were below the lower limit of quantification (LLOQ) in all patients. E2 remained below LLOQ throughout the treatment period in three patients (15%). Persistent E2 elevation above LLOQ was observed in six patients (30%), while isolated E2 elevations occurred in 10 patients (50%). One patient experienced transient E2 elevation in two sporadic measurements. Serum E2 variations were shown by using both LC–MS/MS methods. Vaginal pH, vaginal maturation index (VMI), and VVA symptoms significantly improved during treatment.

**Conclusion:**

Intravaginal estradiol therapy (10ug) during adjuvant letrozole resulted in transient increases in systemic E2 levels among early BC patients with VVA. Highly sensitive LC–MS/MS is a promising method for monitoring E2 levels during aromatase inhibitor (AI) therapy.

## Introduction

Aromatase inhibitors (AIs) are the preferred adjuvant therapy for postmenopausal women with early-stage hormone receptor-positive breast cancer (BC) [1]. Adjuvant endocrine therapy, including AIs, is typically recommended for five to ten years, with extended treatment offered for those with high-risk features [2]. The decrease in estrogen levels during AI therapy leads to changes in the vaginal epithelium, resulting in vaginal dryness and other symptoms of vulvovaginal atrophy (VVA). VVA affects over 50% of postmenopausal women, with a prevalence of up to 75% among BC patients receiving adjuvant endocrine therapy. VVA negatively affects the patients` quality of life (QoL) [3]. VVA symptoms can also reduce treatment adherence [4], and consequently lead to negative effects on survival [5].

The diagnosis of VVA is primarily based on patient-reported symptoms, including vaginal dryness, itching, burning, irritation, pain, dyspareunia or dysuria [6]. Testing vaginal pH and vaginal maturation index (VMI) may support the diagnosis. In VVA, vaginal pH typically exceeds 5.0, leading to *lactobacilli* loss and overgrowth of other bacteria, and an increased risk of urinary tract infections. Histologically, VVA is characterized by reduced elasticity and vascularity, resulting in thin, friable vaginal mucosa and decreased secretion. VMI reflects the proportion of parabasal, intermediate and superficial cells in vaginal tissue, with reduced estrogen correlating with increased parabasal cells, fewer intermediate cells and an absence of superficial cells [7].

While non-hormonal moisturizers and lubricants may offer temporary relief of VVA symptoms, local hormonal treatments are more effective in restoring the vaginal epithelium from its` atrophic state [8–10]. In healthy postmenopausal women, topical estrogen therapy significantly improves vaginal epithelium maturation with minimal systemic effects [8, 11]. However, the safety of local hormonal treatment for VVA in BC patients remains debated due to concerns about systemic estrogen absorption and potential impact on BC recurrence risk.

Two large cohort studies found no significant differences in BC recurrence risk between hormone-receptor positive BC survivors who used local hormonal treatment for VVA and those who did not [12, 13]. Concomitant use of topical vaginal estrogen and tamoxifen does not increase BC recurrence risk [14]. However, the effect of local estrogen levels on BC recurrence risk during AIs remains unclear. Randomized studies have not been performed and observational studies have reported conflicting results. A Danish cohort study suggested an increased BC recurrence risk, though not mortality risk, among patients using vaginal estrogen during adjuvant AI therapy [13]. Further, a recent British cohort study found no increase in BC-specific mortality among patients using concomitant estrogen and AIs [15].

Current data on the effects of concomitant AIs and local estrogens on blood estrogen levels are limited. Existing studies suffer from small sample sizes, differing estrogen preparations, varying estrogen measurement methods and short follow-up periods. Few small studies have reported transient increases in blood estrogen levels in some AI users following topical estrogen use [16–19].

The primary aim of this study was to analyze changes in serum E2 levels using both routine and highly sensitive LC–MS/MS methods in postmenopausal women with early-stage hormone receptor-positive BC undergoing adjuvant letrozole therapy and concomitant vaginal estrogen therapy (Vagifem®) for symptomatic VVA. Additionally, QoL and menopause-related symptoms were monitored during the local estradiol therapy.

## Patients and methods

 The study was conducted between November 2020 and May 2024 at the Comprehensive Cancer Center, Helsinki University Hospital, Finland. Eligible patients were postmenopausal women (> 50 years old) with early-stage hormone receptor-positive BC treated with adjuvant letrozole for at least six months and developed symptoms of AV during this period. Exclusion criteria included premenopausal status, recent use of local estrogen therapy within the previous three months, irregular use of letrozole, and metastatic disease.

VVA diagnosis was based on patient-reported symptoms, with gynecologic examinations, including speculum examination with transvaginal sonography, performed for all participants at baseline. Vaginal Pap smears were used to determine the vaginal maturation index (VMI) by assessing the proportions of parabasal, intermediate and superficial cells in the vaginal wall, along with other cytological findings according to the Bethesda System for cervical cytology. Vaginal pH was measured using litmus paper placed on the vaginal wall until moistened, serving as an indicator of VVA.

Eligible patients were treated with Vagifem® (17β-estradiol hemihydrate) 10 μg vaginal tablets for 12 weeks. Patients were instructed to insert one vaginal tablet daily using the provided applicator for 14 consecutive days, followed by one tablet twice weekly (Monday and Thursday evenings). Blood samples were collected at baseline, prior to the initiation of study treatment, and at two, four, eight, and 12 weeks thereafter, within 24 to 60 h of vaginal tablet administration. Serum was separated by centrifugation within 1 h of collection and the samples were stored at − 80 °C until analysis.

Patients completed the EORTC QLQ-C30 and EORTC QLQ-BR23 questionnaires to assess QoL, with higher scores indicating better QoL or less QoL related symptoms, and the Women`s Health Questionnaire (WHQ), where higher scores indicated greater distress and dysfunction, at baseline and after 12 weeks. Menopause-related symptoms were assessed using a structured 19-item questionnaire [20], while sexual function was evaluated with the McCoy Female Sexuality Questionnaire, which was completed by only half of the patients due to its limitation to sexually active participants. Higher scores on both questionnaires indicated greater severity or frequency of symptoms.

A follow-up gynecologic examination and oncology consultation were conducted at the end of the study to assess the patients` response to topical estradiol therapy. Patients who maintained serum E2 levels below LLOQ (< 5 pmol/L) and benefited from the therapy were considered for continued treatment off-study.

The primary objective was to analyze changes in serum E2 levels using two LC–MS/MS methods during concurrent adjuvant AI therapy and intravaginal estradiol treatment. Serum E2 levels were measured using both a routine LC–MS/MS method (Sciex Citrine™ Triple Quad™ LC–MS/MS system, E2-MS, HUSLAB, LLOQ 10 pmol/L) and a highly sensitive LC–MS/MS method (hsE2-MS, LLOQ 5 pmol/L, CV < 20%, LOD 1 pmol/L). The hsE2-MS method allowed for the detection of very low serum E2 concentrations, even below the LLOQ, within the range of 1–5 pmol/L, providing a precise assessment of the effect of letrozole and vaginal estrogen on E2 levels [21, 22].

The intervention was considered unsuccessful if persistent elevation of E2 (> 5 pmol/L in two consecutive tests) was observed after treatment initiation.

The study protocol was approved by local ethics committee of Helsinki University Hospital on April 20, 2020. Informed consent was obtained from all participants included in the study. The trial is registered in the Helsinki and Uusimaa Hospital District Clinical Trials Register under EudraCT Number 2019–001234-34 and on ClinicalTrials.gov (Trial Registration Number NCT06654570).

## Statistical methods

Data were analyzed using SPSS statistic Version 28. Baseline demographics and patient characteristics were summarized using either median and range or mean and standard deviation, depending on data distribution. The correlation between the two LC–MS/MS-methods was assessed using the Spearman`s correlation coefficient, and differences between the methods were evaluated using the paired *t*-test. Logistic regression analysis was used to determine whether clinical or laboratory measurements at baseline and at two, four, eight, and 12 weeks post-treatment could predict outcomes. *P*-values < 0.05 were considered statistically significant, and all tests were two-sided. The significance of changes in QoL and menopause-related symptoms scores from baseline to 12 weeks was tested using paired t-tests. Given the large number of variables tested, a Bonferroni correction was applied where appropriate, adjusting the significance level to < 0.001.

## Results

### Participants and demographics

Twenty postmenopausal women with hormone receptor-positive early BC were included in the study. The median age of the participants was 66 years (range; 53–77), and they had received adjuvant letrozole for a median duration of two years (range; 0.5–5 years). Most patients (85%) had T1 tumors, and 75% were node-negative. All tumors were ER-positive, and 90% were HER2-negative. Baseline characteristics of the patients are summarized in Table 1.

**Table 1:** Baseline characteristics of the patients (n = 20)

Variable *	
Age (years)	67 ± 6
Weight (kg)	69 ± 10
BMI (kg/m2)	25 ± 4
Waist circumference (cm)	89 ± 13
Vaginal pH	6.7 ± 0.9
Routine E2-MS (pmol/L)	< 10
Sensitive hsE2-MS (pmol/L)	< 1
*Tumor type*	
Ductal	12 (60%)
Lobular	5 (25%)
Other	3 (15%)
*Histological grade*	
1	5 (25%)
2	11 (55%)
3	4 (20%)
*Tumor size*	
T1	17 (85%)
T2	3 (15%)
*Lymph node status*	
N0	15 (75%)
N1	4 (20%)
N2	1 (5%)
*ER status*	
Positive	20 (100%)
*PgR status*	
Positive	14 (70%)
Negative	6 (30%)
*Her-2 status*	
Positive	2 (10%)
Negative	18 (90%)
*M status*	
M0	20 (100%)
Ki-67 (%)	21 ± 20

^*^Mean and SD or number and (%)

### Serum E2 concentrations

Baseline serum E2 levels measured with the highly sensitive LC–MS/MS (hsE2-MS) method were below 5 pmol/L (LLOQ) and even below 1 pmol/L (LOD) in all study patients. When measured with the routine LC–MS/MS-method, baseline serum E2 levels were below 10 pmol/L (LLOQ) in all but one patient, who had a significantly high E2 level of 550 pmol/L.

For one patient, E2 measurements using the hsE2-MS assay were missing at the two- and four-week time points during vaginal estradiol therapy. However, all E2 values measured by the routine E2-MS-assay were available for all patients.

**Fig. 1:**
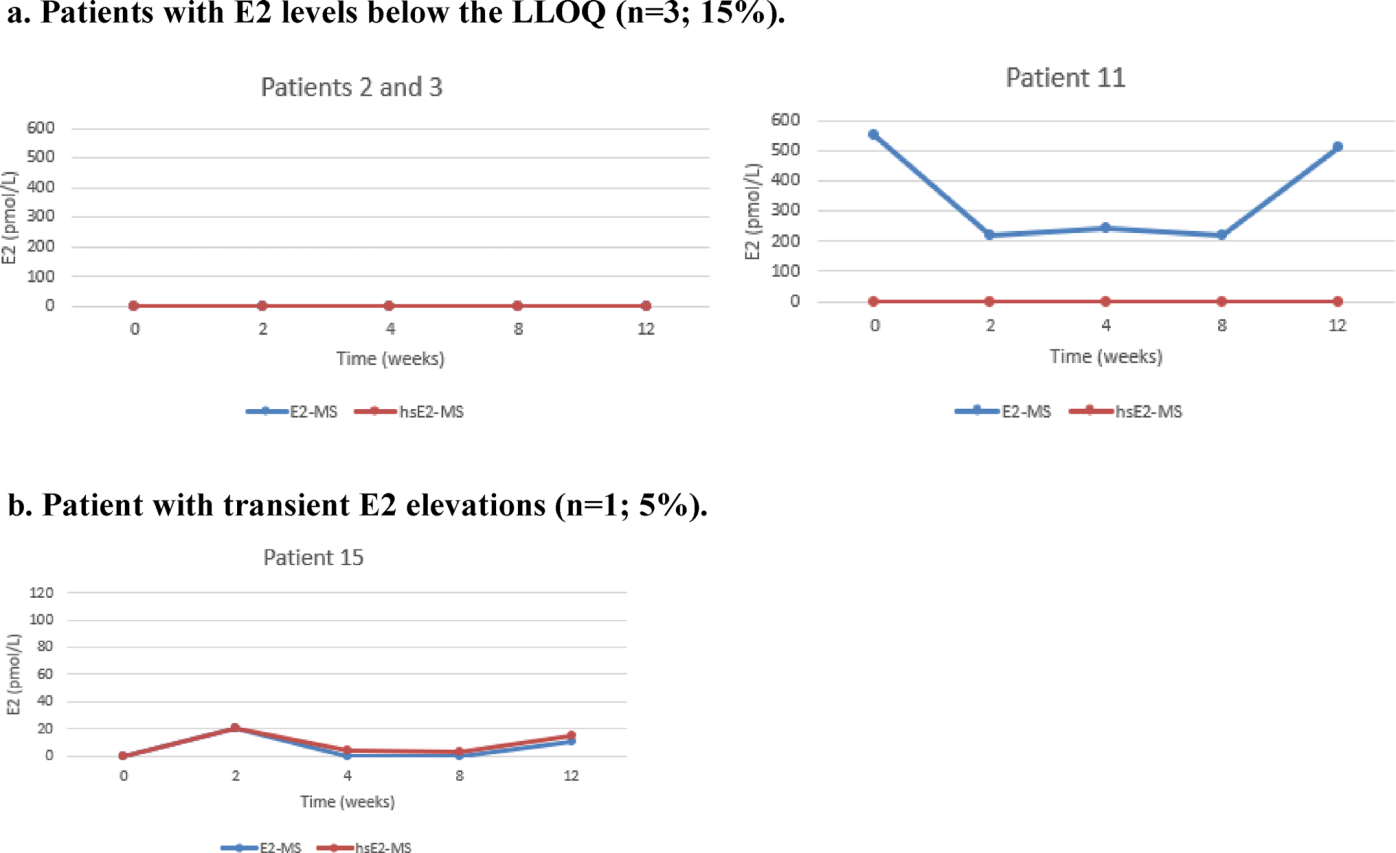

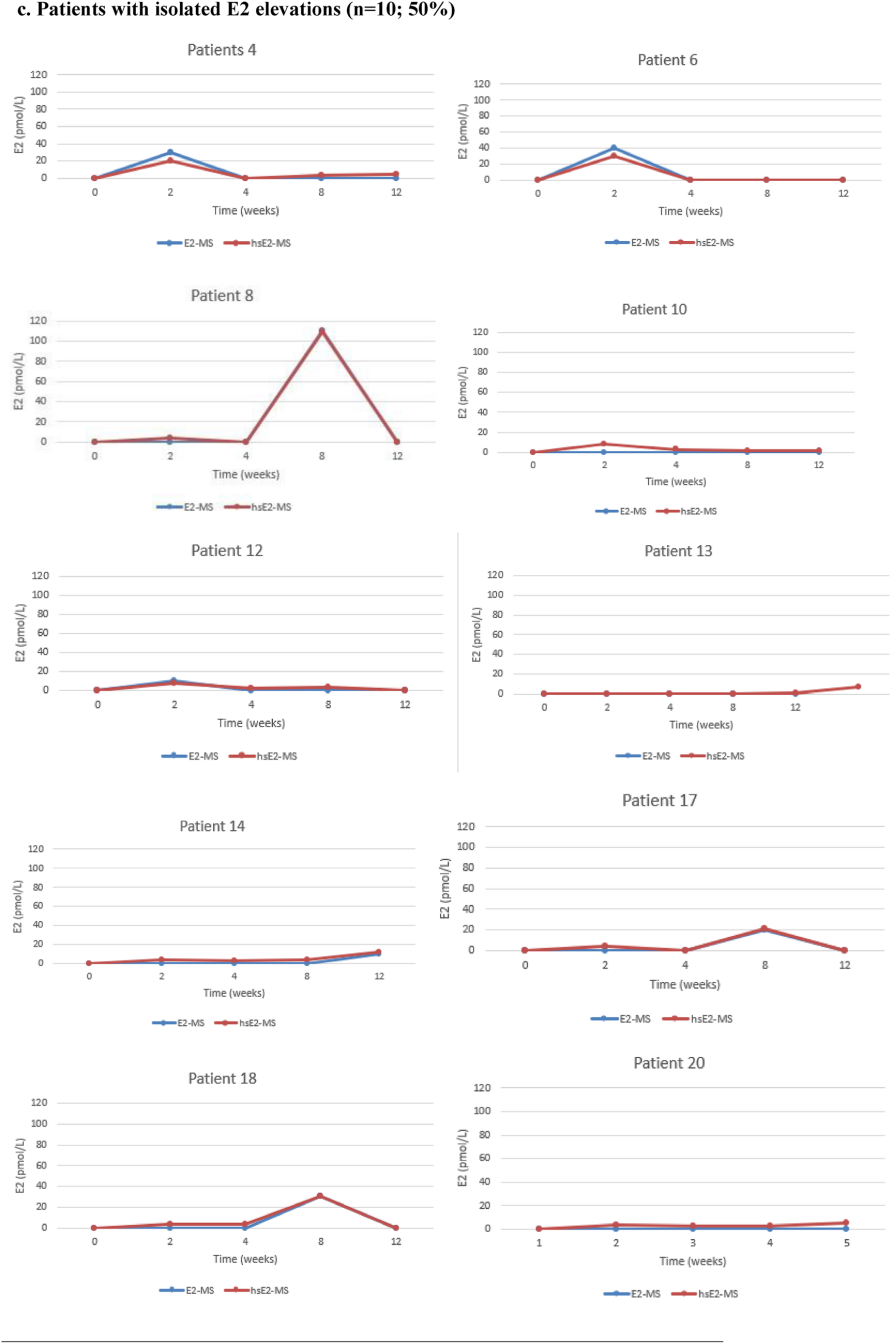

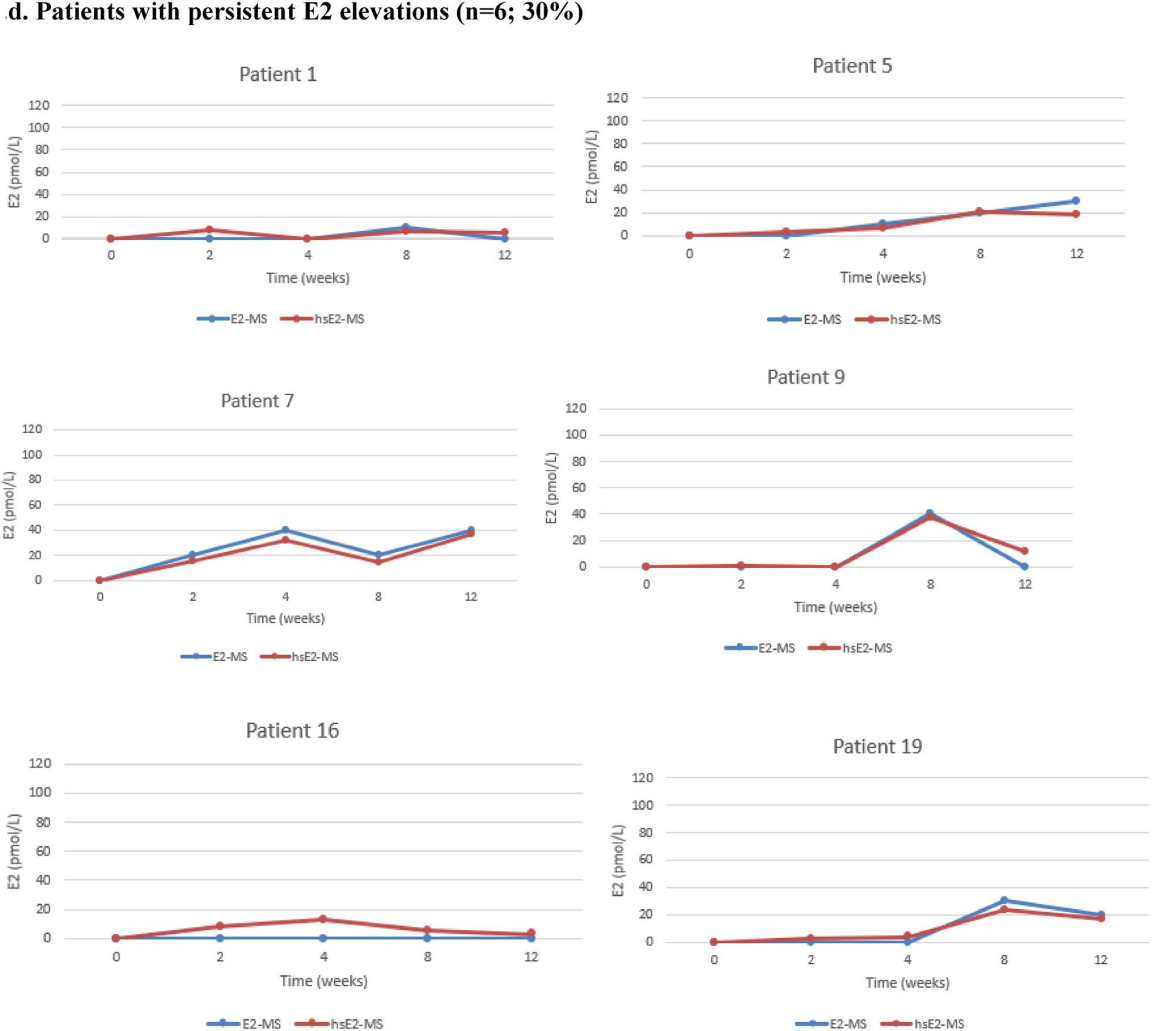
Individual variations in E2 levels for each BC patient treated with concomitant letrozole and vaginal estradiol (Vagifem®) at baseline and at 2, 4, 8 and 12 weeks, measured by two different LC–MS/MS methods. The blue line represents the routine E2-MS method (LLOQ of 10 pmol/L) and the red line the highly sensitive hsE2-MS method (LLOQ of 5 pmol/L). In order to clarify the method comparison all the results below the LLOQs were artificially set at 0 pmol/L of E2 for the both E2-MS and hsE2-MS methods in the Figs. 1a–1d. **a** Patients with E2 levels below the LLOQ (n = 3; 15%). Throughout the study, three patients consistently maintained serum E2 levels below the LLOQ. These patients demonstrated a reliable suppression of E2 during treatment with letrozole and vaginal estrogen therapy. A significant discrepancy was noted in **Patient 11**, whose E2 levels remained below the LLOQ of 5 pmol/L according to all measurements taken with the hsE2-MS method. In contrast, the E2-MS method unexpectedly indicated high E2 levels in this patient. **b** Patient with transient E2 elevations (n = 1; 5%). **c** Patients with isolated E2 elevations (n = 10; 50%) Half of the study participants experienced isolated E2 elevations at various time points during the study. These spikes in E2 levels did not persist and were no consistent over multiple time points. **d** Patients with persistent E2 elevations (n = 6; 30%) Six patients demonstrated persistent E2 elevations across multiple consecutive measurements, indicating a more sustained rise in E2 levels during letrozole and vaginal estrogen therapy

Figure 1 illustrates the individual E2 levels for each patient, measured using both routine and highly sensitive LC–MS/MS methods. After the initiation of Vagifem®, serum E2 levels analyzed by the hsE2-MS method remained below LLOQ (< 5 pmol/l) throughout the study period in three patients (15%). Persistent E2 elevation, defined as two consecutive measurements above the LLOQ, was observed in six patients (30%), with mean E2 levels of 6.2 pmol/L at two weeks, 9.3 pmol/L at four weeks, 18 pmol/L at eight weeks, and 15.5 pmol/L at 12 weeks. Isolated E2 elevations occurred in 10 patients (50%): four patients had E2 elevations at two weeks, ranging from 7 to 30 pmol/L; two patients had elevated E2 levels at eight weeks (30 pmol and 109 pmol/L, respectively); and three patients had elevations at 12 weeks (5 pmol/L, 7 pmol/L and 12 pmol/L, respectively). One patient experienced transient E2 elevation in two sporadic measurements, with E2 rising to 20 pmol/L at two weeks, returning below LLOQ at four and eight weeks, and rising again to 15 pmol/L at 12 weeks.

No significant differences were observed between the mean E2 levels measured by the routine E2-MS method and the hsE2-MS method at any time point, except for one patient. At two weeks, the mean E2 levels were below 10 pmol/L measured with the routine E2-MS method and 7.4 pmol/L (SD 8.2 pmol/L) with the hsE2-MS method (*p* < 0.001). At four weeks, the mean E2 levels were below 10 pmol/l and 3.9 pmol/L (SD 7.6), respectively (*p* < 0.001). At eight weeks, the E2 levels were 14.7 pmol/L (SD 26.5) and 14.3 pmol/L (SD 25.0) (*p* < 0.001), and at 12 weeks, they were below 10 pmol/L and 6.9 (SD 9.4) (*p* < 0.001).

As for the one patient with discrepant E2-levels in Fig. 1a., the baseline serum E2 level was below 5 pmol/L (LLOQ) with the hsE2-MS method, while the routine E2-MS method resulted a high E2 level of 550 pmol/L. Additionally, serum E2-levels with the E2-MS method were 220 pmol/l at two weeks, 240 pmol/l at four weeks, 220 pmol/L at eight weeks, and 510 pmol/L at 12 weeks.

### Vaginal pH

**Fig. 2:**
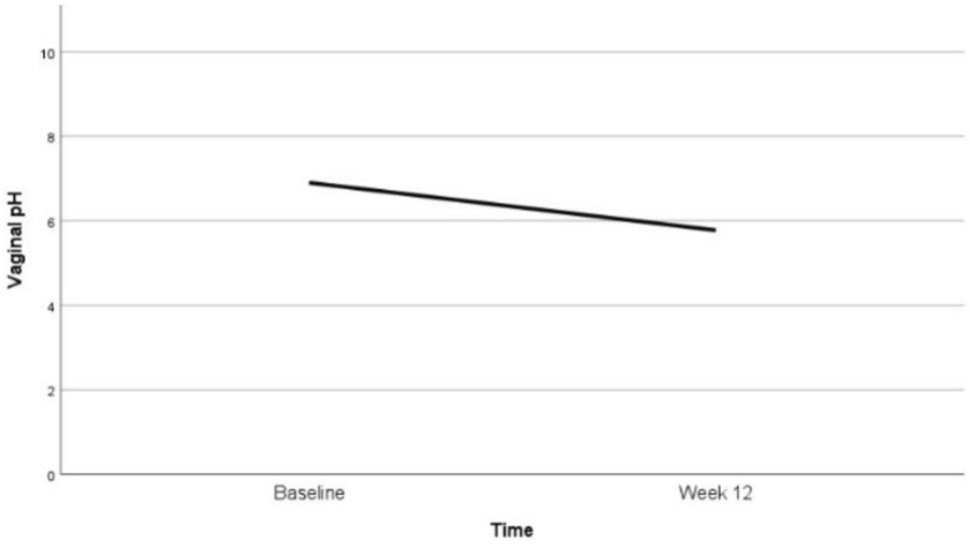
Vaginal pH: changes in mean values at baseline and after 12 weeks of vaginal estradiol treatment

At baseline, 75% of the patients had a vaginal pH of ≥ 7, with a mean of 6.9 (95% CI 6–8), indicating severe vaginal atrophy. Additionally, inflammatory changes were observed in 11 patients. After 12 weeks of topical estradiol therapy, 90% of patients exhibited a shift towards a more acidic vaginal environment, with a mean pH of 5.8 (95% CI 5–6), indicating mild atrophy and no signs of inflammation. The change in mean vaginal pH from baseline to 12 weeks was statistically significant (*p*-value < 0.001), as shown in Fig. 2.

### Vaginal maturation index (VMI)

**Fig. 3:**
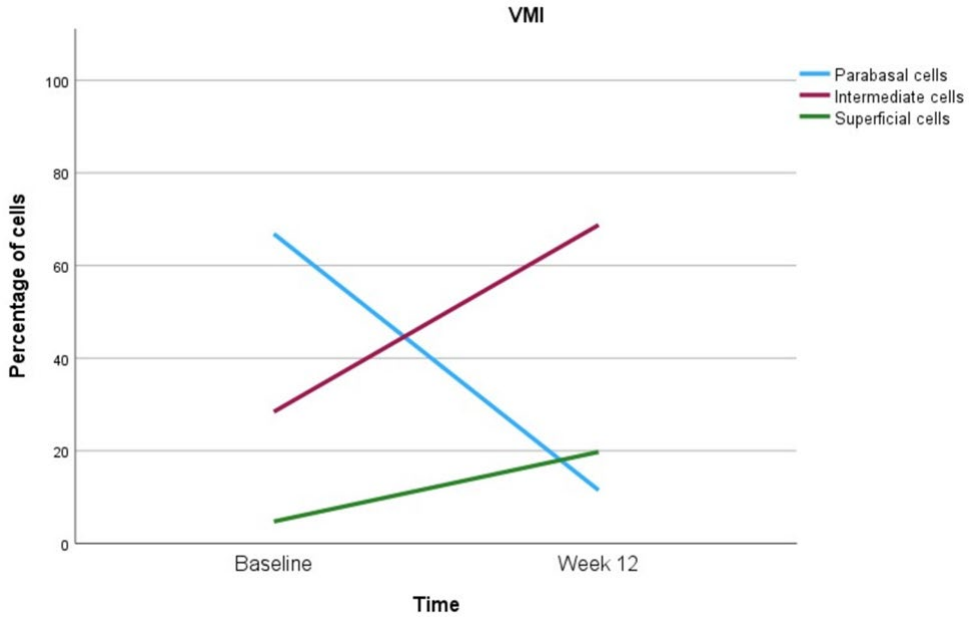
VMI: changes in cell types at baseline and after 12 weeks of vaginal estradiol treatment

The proportion of parabasal cells decreased from 67% ± 38 at baseline to 12% ± 23 at 12 weeks (*p-*value < 0.001). Conversely, the proportion of intermediate cells increased from 28% ± 32 at baseline to 68% ± 23 at 12 weeks (*p-*value < 0.001). Superficial cells, which were initially 5% ± 9 at baseline, increased modestly to 20% ± 14 at 12 weeks (*p*-value < 0.001). Changes in the proportions of these cell types over time are shown in Fig. 3.

### Quality of life

QoL and symptom measures were assessed at baseline and after 12 weeks of vaginal estradiol treatment. Mean QoL scores, as measured by the EORTC QLQ-C30 and EORTC QLQ BR-23 questionnaires, are presented in Tables 2 and 3. The global health status, functional scores and symptom scores from the EORTC QLQ-C30, as well as the functional and symptom scales from the EORTC QLQ BR-23, remained stable throughout the study. Analysis of the WHQ revealed significant reductions in somatic symptoms (e.g. headaches, backaches, pins and needles), as well as a decrease in anxiety or fears (Table 4). According to the structured questionnaire assessing 19 key menopausal symptoms, there was a significant reduction in vaginal dryness (p < 0.001) during vaginal estradiol treatment (Table 5). Sexual function, assessed using the McCoy Female Sexuality Questionnaire, also improved, with reductions in issues like insufficient vaginal lubrication and painful sexual intercourse reported during the treatment period (Table 6).

**Table 2:** EORTC QLQ-C30 scores at baseline and after 12 weeks

Measure	(n)	Baseline Mean ± SD	12 weeks Mean ± SD	*P* value
*Global health scale*				
Global health status/QoL	20	70 ± 12	69 ± 18	1.000
*Functioning scales*				
Physical functioning	20	85 ± 15	85 ± 15	1.000
Role functioning	19	87 ± 22	89 ± 23	0.707
Emotional functioning	20	87 ± 12	86 ± 13	0.630
Cognitive functioning	20	91 ± 16	89 ± 14	0.667
Social functioning	20	95 ± 15	95 ± 11	1.000
*Symptom scales*				
Fatigue	20	21 ± 18	25 ± 20	0.439
Nausea and vomiting	20	0	4 ± 9	0.104
Pain	20	23 ± 26	28 ± 25	0.508
*Symptom single items*				
Dyspnea	20	3 ± 10	11 ± 16	0.042
Insomnia	20	38 ± 20	28 ± 30	0.069
Appetite loss	20	2 ± 7	4 ± 11	0.331
Constipation	20	13 ± 23	4 ± 11	0.030
Diarrhea	20	7 ± 17	5 ± 17	0.578
Financial difficulties	20	7 ± 23	9 ± 27	0.331

Values are expressed as mean ± SD

Bonferroni-corrected significance level < 0.001

**Table 3:** EORTC QLQ-BR23 scores at baseline and after 12 weeks

Measure	(n)	Baseline (n) Mean ± SD	12 weeks Mean ± SD	*P* value
*Functioning scales*				
Body image	20	81 ± 21	83 ± 18	0.461
Sexual functioning	19	81 ± 18	74 ± 17	0.100
Sexual enjoyment	10	63 ± 29	48 ± 18	0.025
Future perspective	20	62 ± 22	61 ± 25	1.000
*Symptom scales*				
Systemic therapy symptoms	20	26 ± 16	25 ± 19	0.447
Breast symptoms	20	9 ± 8	11 ± 13	0.445
Arm symptoms	20	10 ± 14	13 ± 14	0.319
Upset by hair loss	10	44 ± 37	43 ± 39	0.760

Values are expressed as mean ± SD

Bonferroni-corrected significance level < 0.001

**Table 4:** Scores for the Women`s Health Questionnaire (WHQ) at baseline and after 12 weeks

Women`s heath questionnaire factor	(n)	Baseline Mean ± SD	12 weeks Mean ± SD	*P* value
Vasomotor symptoms	18	0.28 ± 0.43	0.69 ± 0.42	0.031
Somatic symptoms	18	0.67 ± 0.26	0.31 ± 0.23	**0.001**
Anxiety and fears	18	0.81 ± 0.25	0.19 ± 0.36	** < 0.001**
Depression	18	0.52 ± 0.14	0.49 ± 0.12	0.545
Sleep problems	18	0.50 ± 0.26	0.52 ± 0.33	0.883
Sexual behavior	18	0.25 ± 0.33	0.63 ± 0.36	0.011
Memory and concentration	18	0.59 ± 0.41	0.44 ± 0.41	0.346
Attractiveness	18	0.35 ± 0.27	0.60 ± 0.31	0.015

Values are expressed as mean ± SD

Bonferroni-corrected significance level < 0.001

**Table 5:** Prevalence of menopause-related symptoms at baseline and after 12 weeks, measured by a structured questionnaire for leading 19 menopausal symptoms

Menopause-related symptoms	(n)	Baseline Mean ± SD	12 weeks Mean ± SD	*P* value
1Night sweats	18	2.6 ± 1.3	2.5 ± 1.3	0.607
2Hot flushes	18	3.3 ± 1.1	2.8 ± 1.4	0.046
3Numbness	18	2.4 ± 1.3	1.9 ± 1.2	0.028
4Insomnia	18	3.3 ± 0.7	3.0 ± 0.8	0.135
5Irritability	18	2.4 ± 1.0	2.2 ± 0.9	0.260
6Feeling exhausted	18	2.2 ± 0.6	2.2 ± 0.8	0.749
7Depressive mood	18	1.7 ± 0.9	1.6 ± 0.9	0.834
8Dizziness	18	1.5 ± 0.7	1.3 ± 0.6	0.269
9Weakness	18	1.7 ± 0.7	1.6 ± 0.9	0.631
10Aching joints or muscles	18	3.1 ± 1.1	2.8 ± 1.2	0.135
11Headache	18	2.1 ± 1.0	1.9 ± 0.9	0.607
12Palpitation	18	1.9 ± 1.1	1.8 ± 0.9	0.542
13Vaginal dryness	18	3.3 ± 1.0	1.9 ± 1.1	** < 0.001**
14Oedema	18	2.0 ± 1.1	1.7 ± 1.0	0.250
15Shortness of breath	18	1.3 ± 0.6	1.1 ± 0.3	0.269
16Dryness of mouth	18	2.9 ± 1.2	2.8 ± 1.2	0.790
17A feeling of a lump in the throat	18	1.6 ± 1.0	1.1 ± 0.3	0.057
18Nausea	18	1.2 ± 0.4	1.2 ± 0.5	1.000
19Trembling	18	1.2 ± 0.7	1.1 ± 0.5	0.790

Values are expressed as mean ± SD

Bonferroni-corrected significance level < 0.001

**Table 6:** Sexual function at baseline and after 12 weeks, measured with McCoy Female Sexuality Questionnaire

	(n)	Baseline Mean ± SD	12 weeks Mean ± SD	*P*-value
Insufficient vaginal lubrication	10	5.6 ± 1.6	3.9 ± 2.1	**0.016**
Painful sexual intercourse	10	5.3 ± 2.2	3.5 ± 2.5	**0.012**
Sexual satisfaction	15	3.4 ± 1.2	3.6 ± 1.3	0.284

Values are expressed as mean ± SD

## Discussion

In postmenopausal women, circulating E2 levels typically remain below 30 pmol/L, depending on the measurement method used [23–26]. AIs further reduce E2 levels to below 1-3 pmol/L by inhibiting the aromatase enzyme by more than 97 % [27]. Measuring these low E2 levels requires highly sensitive methods, and advances in mass spectrometry-based techniques have improved the detection capabilities of these concentrations [28]. However, the implications of such low E2 levels on BC recurrence risk remain unclear [29].

In this study, baseline serum E2 levels in all 20 patients treated with letrozole were below the LLOQ of 5 pmol/L when measured using the hsE2-MS method. No peaks corresponding to all quantifier ions were identified in the MS chromatograms, even at our LOD of 1 pmol/L with the hsE2-MS method, which is consistent with previous findings [22]. Baseline E2 levels in 19 of 20 patients were also below the LLOQ of 10 pmol/L when measured with the routine E2-MS method.

Detecting minor E2 fluctuations during concurrent AI and local estrogen treatment is challenging due to such low E2 levels, often below 3 pmol/L [30]. Therefore, two LC-MS/MS methods were used. They showed a strong correlation with each other, except for one patient. In this case, the baseline serum E2 level was below 5 pmol/L (LLOQ) when measured with the hsE2-MS method, while the routine E2-MS method resulted a high E2 level of 550 pmol/L. Throughout all analyses for this patient, the E2 levels measured by the routine E2-MS were significantly different from those obtained using the hsE2-MS method. In contrast, levels measured with the hsE2- MS method remained below the LLOQ of 5 pmol/L throughout the study. Similar issues have been shown in LCMS/ MS methods using short columns and fast run times, which can lead to matrix effects or insufficient resolution of analytes in HPLC, as well as challenges in the quantitation of steroid ions. Interfering peaks caused by nonsteroidal drugs taken by the patient or other endogenous interference factors have been also reported [31, 32]. In this particular case, no definitive cause was identified, as there were no serum samples remaining from the routine E2-MS method for further investigation.

Using the hsE2-MS method, serum E2 levels remained consistently below the LLOQ throughout the study in three patients (15 %), demonstrating consistent estrogen suppression below 5 pmol/L during concurrent letrozole and vaginal estradiol therapy. Among the remaining 17 patients with elevated E2 levels, mean E2 levels increased to 8.8 pmol/L (range <5 to 30 pmol/L) at two weeks, decreasing to 4.6 pmol/L (range <5 to 32 pmol/L) after four weeks of therapy. The highest mean E2 elevation occurred at eight weeks, reaching a mean of 16.8 pmol/L (range <5 to 109 pmol/L), followed by a decrease to 8.1 pmol/L (range <5 to 37 pmol/L) at 12 weeks. Despite these individual fluctuations, the E2 levels remained within the postmenopausal range for most patients.

Previous studies suggest that vaginal maturation during local estrogen therapy might increase systemic absorption initially, which may stabilize over time. However, AI therapy may impair complete mucosal repair, leading to fluctuating E2 levels during maintenance treatment. A recent systemic review [24] highlighted that estrogenabsorption varies by dose, formulation, and vaginal placement. The threshold of circulating E2 that increases BCrecurrence risk is still unknown, and the impact of low-dose estrogen absorption on BC outcomes is also unclear.Although circulating estrogen levels are commonly used to assess treatment efficacy in AI-treated patients, the relevance of estrogen levels has been questioned. Estrogen concentrations in BC tissue are significantly higher than in plasma, and no correlation has been observed between plasma and BC tissue estrogen levels [33]. This discrepancy is thought to result from differential uptake from circulation and surrounding benign breast tissue, as well as localized estrogen within breast tissue.

Meta-analyses [34, 35] have shown no significant systemic absorption of estrogen from local estrogen treatments in BC patients receiving adjuvant AIs. Several studies have demonstrated that vaginal estradiol effectively alleviates symptoms and improves vaginal mucosa and pH in postmenopausal women with VVA [36, 37]. However, it has remained unclear whether local estrogens can repair the vaginal mucous membranes in women undergoing AI therapy. In this study, significant improvements in vaginal pH, along with improvements in superficial cells and intermediate cell, and reduced parabasal cells from baseline to 12 weeks were significant. Most patients showed only mild atrophy without inflammatory changes at the end of the treatment period. These improvements were statistically significant in both clinical examinations and patient-reported symptoms.

In some AI users, the severity of VVA drives patients to use local estrogens despite potential risks. A wellconsidered application of low-dose local estrogen may pose a lower risk of BC recurrence compared to discontinuation of AI therapy due to adverse effects. Therefore, intravaginal estrogen therapy for AI-induced VVA in BC survivors should be customized according to the patient’s risk/benefit profile and clinical presentation. This personalized approach ensures a balance between the need to alleviate VVA symptoms and the critical goal of maintaining the efficacy of AI therapy in reducing the risk of BC recurrence.

This study has several limitations, including its small sample size and a lack of long-term follow-up to assess the prolonged effects of local estrogen on systemic E2 levels and BC recurrence risk. Inter-individual variability in E2 levels also complicates conclusions on systemic effects of Vagifem ®. Furthermore, the timing of blood sample collection may have influenced E2 absorption measurements by capturing an early spike, potentially leading to an overestimation of initial absorption. However, this does not fully explain the highest E2 elevations observed at eight weeks, suggesting additional factors influencing E2 fluctuations. Finally, only sexually active participants completed the McCoy Female Sexuality Questionnaire, limiting coverage of data on sexual function across the cohort.

The strengths of this study include the use of a highly sensitive LC-MS/MS method, enabling precise detection of very low E2 levels, along with comprehensive symptom evaluations, including objective measures like vaginal pH, VMI, and assessment of QoL and menopause-related symptoms. Further research is needed to clarify the relationship between minor E2 fluctuations and BC recurrence risk and to determine the value of monitoring low serum E2 levels during concurrent AI and local estrogen therapy to optimize treatment outcomes.

## Conclusion

Intravaginal estrogen therapy with 10 ug estradiol tablets during adjuvant letrozole therapy led to transient increases in systemic E2 levels in postmenopausal BC patients with VVA. Persistent E2 elevation was observed in 30% of patients, while 15% maintained serum E2 levels below the LOD of 1 pmol/L throughout the study. Vaginal pH, VMI, and VVA symptoms showed significant improvement during the treatment period. The use of highly sensitive LC–MS/MS is a promising method for monitoring E2 levels during AI therapy.

## Data Availability

The datasets generated and/or analyzed during the current study are not publicly available due to the restrictions outlined in the consent form. However, they are available from the corresponding author on reasonable request.
